# Foreign Correspondence

**Published:** 1872-04-01

**Authors:** 


### Foreign Correspondence.

TUBINGEN, WURTEMBERG, GERMANY,
March 25th, 1872.

The University of Wurtemberg, of which this picturesque little village is the seat, is probably best known to the profession in America as the home of the late lamented Prof. von Niemeyer, and for many years the seat of his labors. As the leading member of their medical faculty, his loss is, of course, severely felt by the institution. One of the oldest universities in Germany, it has always been noted mainly for its theological school. Its medical department, however, offers special advantages for study in certain departments, more especially in the elementary branches of anatomy, physiology, chemistry, etc., the lectures being much less crowded, and the opportunities for personal observation and instruction much superior, to those offered in larger schools of Vienna or Berlin. Like the schools at Bonn, Heidelberg, etc., it labors, however, under the very great disadvantage of being situated in a small village, where the opportunities for hospital or clinical instruction are necessarily very limited. Their students are consequently obliged to resort to the hospitals of some of the larger cities for their final studies.

Prof. Liebermeister, the successor of Prof. Niemeyer, delivered his first course in Tubingen during the past semister. He is spoken of by the students as being a very interesting lecturer.

In an article lately published by Professor Liebermeister on the treatment of typhoid fever, I notice that he advocates a course of treatment by cold baths, to the exclusion of almost all medication. The patient is placed in a general bath of moderately cold water, and after remaining for an instant, is dried off and returned to bed. These baths are repeated more or less frequently, according to the amount of fever, heat and dryness of skin, etc. He claims that under this system the mortality is very considerably diminished. The idea is not original, however, with Prof. Liebermeister. Experiments were made with the same course of treatment, some time ago, in several of the English hospitals, but with very unfavorable results.

On our journey up the Rhine we paused for a day at Bonn, to visit the University there—the school with which the well-known pathologist and histologist, Prof. Rindfleisch, is connected. Unfortunately, however, both there and at Heidelberg, we arrived just at the commencement of the Easter vacation, when many of the Professors and most of the students were away traveling or visiting friends, so that there was little beyond the bare University walls to be seen. At Heidelberg, new and extensive hospital buildings have been for some time in course of erection. When completed, if the wards can be filled with patients, it will be a great addition to the value and efficiency of their school. While there I had the pleasure of meeting the celebrated chemist, Prof. Bunson, and also of inspecting his laboratory, probably the most complete and extensive in Europe.

In glancing over the catalogues of the German Universities, there is one thing that strikes a stranger as being somewhat novel, namely, the fact that they include in the list of the Faculty, Professors of riding, fencing, gymnastics, swimming, and last, but not least, of dancing. Whether proficiency in all of these accomplishments is necessary to a proper mental culture may well be doubted, but on the principle of affording proper facilities for thorough physical training and development, the German schools are certainly right· It is a department the value and importance of which is too little appreciated in our Universities.

We feel that an apology is due for the general dearth of news in this letter. Being merely on our journey as yet up toward that present supposed center of all medical knowledge and discovery, Vienna, we have here recorded merely the wayside gleanings, as it were, and send them on chiefly in the way of reporting progress. F. H. D.

